# Interaction Between Sulfur and Iron in Plants

**DOI:** 10.3389/fpls.2021.670308

**Published:** 2021-07-20

**Authors:** Stefania Astolfi, Silvia Celletti, Gianpiero Vigani, Tanja Mimmo, Stefano Cesco

**Affiliations:** ^1^Department of Agricultural and Forestry Sciences (DAFNE), University of Tuscia, Viterbo, Italy; ^2^Department of Life Sciences and Systems Biology, Università degli Studi di Torino, Turin, Italy; ^3^Faculty of Science and Technology, Free University of Bozen-Bolzano, Bolzano, Italy; ^4^Competence Centre for Plant Health, Free University of Bozen-Bolzano, Bolzano, Italy

**Keywords:** iron, sulfur, interaction, methionine, nicotianamine, citrate

## Abstract

It is well known that S interacts with some macronutrients, such as N, P, and K, as well as with some micronutrients, such as Fe, Mo, Cu, Zn, and B. From our current understanding, such interactions could be related to the fact that: (i) S shares similar chemical properties with other elements (e.g., Mo and Se) determining competition for the acquisition/transport process (*SULTR* transporter family proteins); (ii) S-requiring metabolic processes need the presence of other nutrients or regulate plant responses to other nutritional deficiencies (S-containing metabolites are the precursor for the synthesis of ethylene and phytosiderophores); (iii) S directly interacts with other elements (e.g., Fe) by forming complexes and chemical bonds, such as Fe-S clusters; and (iv) S is a constituent of organic molecules, which play crucial roles in plants (glutathione, transporters, etc.). This review summarizes the current state of knowledge of the interplay between Fe and S in plants. It has been demonstrated that plant capability to take up and accumulate Fe strongly depends on S availability in the growth medium in both monocots and dicot plants. Moreover, providing S above the average nutritional need enhances the Fe content in wheat grains, this beneficial effect being particularly pronounced under severe Fe limitation. On the other hand, Fe shortage induces a significant increase in the demand for S, resulting in enhanced S uptake and assimilation rate, similar to what happens under S deficiency. The critical evaluation of the recent studies on the modulation of Fe/S interaction by integrating old and new insights gained on this topic will help to identify the main knowledge gaps. Indeed, it remains a challenge to determine how the interplay between S and Fe is regulated and how plants are able to sense environmental nutrient fluctuations and then to adapt their uptake, translocation, assimilation, and signaling. A better knowledge of the mechanisms of Fe/S interaction might considerably help in improving crop performance within a context of limited nutrient resources and a more sustainable agriculture.

## Introduction

The global human population is expected to increase to 9.7 billion by 2050 and 11.2 billion by 2100 (UN 2015). Thus, providing food and feed in an equitable, healthy, and sustainable manner is a key challenge (Beddington, 2010). The access to adequate and nutritious food is essential to human wellbeing, but to reach this objective global food production needs to be increased by 70% within 2050. Healthy soil is a major factor for agriculture production, but soil resources are finite and non-renewable over the human time scale. Indeed, both natural events and mostly anthropogenic activities (e.g., deforestation, drainage, tillage, etc.) led to soil degradation and consequently to limited access to high-quality soil for provisioning of essential ecosystem services.

This negative trend is expected to continue at least until 2030 (Report: EU agricultural outlook 2017-30). Consequently, to maximize ecosystem services like food production, it is crucial to also recover the marginal agricultural lands for agriculture use. However, they have already been affected by the already existing problems of soil fertility (e.g., nutrient depletion, water scarcity, acidity, salinization, depletion of organic matter, and poor drainage) ascribable to the land-use changes (Scherr, 1999; Tilman et al., 2002; Lal, 2015), which have been often exacerbated by climate change (Pilbeam, 2015). It should also be highlighted that a more massive use of the current farming practices to meet the challenge of more food demand is very likely to lead to more intense competition for natural resources, increased greenhouse gas emissions, and further deforestation and land degradation. This condition is even more dramatic when comparing agricultural productivity between high-income and low-income countries.

The availability of nutrients in different edaphic conditions as well as its effect on the whole plant development and metabolism have been widely described, mainly at the level of a single nutrient at a time. However, at the field scale multiple deficiencies and/or nutrient interactions are very likely to occur. Furthermore, it is well recognized that the deficiency or excess of a single nutrient is typically coordinated with a change in the demand for another, or even more than one, nutrient. In this context, we can distinguish between positive/synergic interaction (when the plant grows better with combined nutrients with respect to the sum of their individual effects) and negative/antagonistic interaction (combining nutrients results in worsening plant growth with respect to the sum of their individual effects) (Fageria, 2001; Marschner, 2012; Pii et al., 2015; Rietra et al., 2017; Xie et al., 2021).

However, although the comprehension of the multi-level interactions among the various mineral elements is considered crucial to understanding the different sensing and signaling pathways induced by a single or multiple shortage/s, the impact of these nutrients’ interactions on crop performance are largely unknown. This lack is certainly ascribable to the complexity of the phenomenon. In fact, the consequences of multiple deficiencies are almost never the mere sum of those caused by each of the individual deficiencies. An example is represented by the interactions between nitrogen (N) and sulfur (S). In this specific case, it has been widely demonstrated that grain productivity benefits from simultaneous N and S fertilization (Kalmbacher et al., 2005; Habtegebrial and Singh, 2006; Mathot et al., 2009; Salvagiotti et al., 2009), most likely for their role in protein synthesis (Crawford et al., 2000). In this respect, the coordination of N and S assimilatory pathways in plants corroborate this hypothesis, suggesting the functionality of putative co-regulation mechanisms (Koprivova et al., 2000). However, when one of the two nutrients is missing, the lacking one represses the assimilation of the other and induces physiological changes aiming at re-balancing the contents in the plant (Hawkesford and De Kok, 2006). Clear links have been also established between S and phosphorus (P) in the soil/plant system. Plants have developed tightly controlled mechanisms to coordinate S transport and homeostasis with photosynthesis and the carbon status, in a similar manner to the inorganic P transport system (Lejay et al., 2008). Plants maintain intracellular homeostasis of both elements in response to their respective external availability. For instance, plant cells operate a rapid replacement of sulfolipids by phospholipids under S deficiency, and *vice versa* during P deficiency (Sugimoto et al., 2010). Such a metabolic switch is evidence of P/S nutritional interdependency.

This review summarizes the current state in the field of the interplay between iron (Fe) and S in plants toward a vision of a more sustainable use of the soil resource and within the context of the great challenge of food security.

## Basis of the Interaction Between Sulfur and Iron

Over recent years, S deficiency has become widespread in many regions of the world. The occurrence of S deficiency has been described in cereals as well as in other crops. The reasons behind this trend are mainly the strong decrease in the inputs of S from atmospheric deposition and the use of high-analysis low S fertilizers (Zhao et al., 1999). At the beginning of the 1980s, environmental policies drastically reduced SO_2_ emissions with a further lowering in the 1990s (Haneklaus et al., 2008), causing a diminished S input to soil. This phenomenon has been worsened using high-analysis low-S fertilizers and the declining use of S-containing fungicides with the final result of a widespread S deficiency for crops (McGrath and Zhao, 1995; McGrath et al., 1996).

Iron deficiency is one of the major agricultural problems leading to lower crop yields and Fe fertilization management has been the focus of attention for the last decades (Kim and Guerinot, 2007; Briat, 2009). With respect to its metabolic roles, Fe nutrient is crucial for the proper functioning of metabolic processes related to electron transport, such as respiration and photosynthesis, as well as for chlorophyll biosynthesis (Briat et al., 2007). Indeed, Fe deficiency firstly appears with a reduced growth and leaf chlorosis in young leaves and then on older ones associated with alteration of the main metabolic pathways. Iron deficiency provokes serious imbalances in the ultrastructure and functionality of chloroplasts, with 90% of Fe present in a leaf localized in the chloroplasts (Morrissey and Guerinot, 2009; López-Millán et al., 2016).

In soils, Fe is present in huge amounts, being the fourth most abundant element in the earth’s crust in percentage after oxygen, silicon, and aluminum. Therefore, widespread limited availability of Fe for plant nutrition is not related to its absolute content into the soil, but rather to its limited solubility. In particular, Fe deficiency is a typical feature of alkaline soils (Marschner, 2012), which cover more than 25% of the earth’s surface (FAO, 2015). Low Fe availability in calcareous soils can be ascribed to an extremely low solubility of soil Fe. Further, alkaline conditions may also depress or even block Fe uptake mechanism from the apoplast into the symplast, which can be related to the pH of the former (Mengel, 1994; Nikolic and Kastori, 2000).

## Sulfur Availability Affects Plant Capability to Cope With Iron Shortage

When Fe is limited in the substrate, plant roots rely on two main strategies to acquire it, i.e., Strategy I and Strategy II, based on Fe^3+^-reduction and Fe^3+^-chelation, respectively (Kobayashi and Nishizawa, 2012). Strategy I, used by all except graminaceous plants, involves: (i) the mobilization of Fe^3+^ ions from soil particles through rhizosphere acidification, likely driven by an increase in plasma membrane H^+^-ATPase activity; (ii) the induction of a ferric chelate reductase (FCR) activity, which allows higher reduction rate of Fe^3+^ to Fe^2+^; and (iii) the uptake of the resulting Fe^2+^ *via* an Fe^2+^ transporter (IRT). The Strategy II system is restricted to grasses, which secrete mugineic acid (MA) family phytosiderophores (PS) from their roots to chelate and solubilize Fe^3+^ in the rhizosphere (Takagi, 1976). The Fe^3+^–PS complexes are then taken up by root cells through the action of Yellow Stripe 1 (YS1) proteins (Murata et al., 2006).

Both mechanisms undoubtedly improve Fe acquisition by plants, although Fe deficiency issues can still occur, regardless of whether the plant is being grown in nutrient-rich or poor soils. In fact, it has been observed that Fe deficiency could be caused by factors other than limited available Fe and, on the other hand, Fe deficiency could be overcome by factors besides Fe supply.

Research on the S/Fe interaction started from the evidence that S-deficient plants showed a limited ability to accumulate Fe. Literature on this topic was minimal. In fact, to our knowledge, one of the first studies on barley plants showed that sulfate availability in Fe-deficient growth medium could affect PS accumulation in root tissues and the extent of ^14^C glucose incorporation into PSs (Kuwajima and Kawai, 1997). Since then, significant interactions between external S supply and Fe homeostasis have been described in several crops, both grasses (Astolfi et al., 2003, 2006a, 2018; Bouranis et al., 2003; Zuchi et al., 2012; Ciaffi et al., 2013; Wu et al., 2015, 2020; Celletti et al., 2016a) and dicots (Zuchi et al., 2009, 2015; Muneer et al., 2014; Paolacci et al., 2014; Coppa et al., 2018), suggesting its independence of the adaptive responses activated by the plant species of both strategies.

Early physiological evidence showed that maize plants exposed to S deficiency had a lower shoot Fe content than those grown in the presence of S (Astolfi et al., 2003; Bouranis et al., 2003). Further, it has been demonstrated in barley that S deficiency could potentially prevent Fe accumulation in shoots by lowering the release rate of PS (Astolfi et al., 2006a) and/or by hindering the capability to take up Fe from the external solution (Astolfi et al., 2006b). In addition, *HvYS1* expression, the specific transporter of Fe^3+^-PS complexes, is modulated by S supply (Astolfi et al., 2010) in barley, suggesting that S mainly affects the Fe acquisition step.

Interestingly, barley plants fully recovered their capability to cope with Fe shortage after resupplying S to S-deficient plants (Astolfi et al., 2010, 2012). After the S resupply, the increase in PS release in root exudates was evident after 24 h of growth in S-sufficient nutrient solution and the increase reached values up to four-fold higher than control after 48 h from S resupply (Astolfi et al., 2010, 2012). A significant drop of Fe accumulation induced by S deficiency has been gathered in durum wheat (Ciaffi et al., 2013) and, later, in rice plants (Wu et al., 2015). Interestingly, transcriptomic analysis of durum wheat roots has identified 377 differentially expressed transcripts under S deficiency (Zamboni et al., 2017). Among them, several transcripts encoding formate dehydrogenase were downregulated by S deficiency, resulting in limited removal of the formate, which is released by the methionine (Met) cycle. As a result, the amount of NADH produced is not sufficient for PS biosynthesis, which is most likely hindered (Mori, 1999; Zamboni et al., 2017). On the other hand, it has been recently shown that S-deficiency-induced reduction of Fe content in rice shoots was associated to a decreased nicotianamine (NA) level, suggesting that S is not only crucial for Fe acquisition but also for its translocation to the shoot (Wu et al., 2020), being NA the main Fe chelator involved in both xylem and phloem Fe transport within the plants (Von Wirén et al., 1999; Briat et al., 2007).

Overall, this evidence indicated that plants require an adequate S supply to efficiently cope with Fe starvation. Thus, it was not surprising that an over-supply of S allowed to improve, specifically in durum wheat, the Fe use efficiency of plants and the accumulation of this micronutrient in plant tissues (Zuchi et al., 2012; Celletti et al., 2016a). Moreover, providing S above adequate concentrations may result in the improvement of wheat Fe use efficiency (Hawkesford et al., 2014) and this S nutritional effect seems to be especially advantageous for plants grown under severe Fe limitation, leading to a significant recovery of deficiency symptoms (Zuchi et al., 2012). However, the positive effect of super-optimal S supply in improving the capability of wheat plants to accumulate Fe was later confirmed in wheat but not in barley and maize plants. In these an antagonistic effect between Fe deficiency and S surplus has been observed, resulting in a reduction of Fe accumulation in shoots (Celletti et al., 2016a). The differential Fe accumulation pattern could most likely be ascribed to the different capability to release PS by the three grasses (Marschner, 2012). Actually, the increased ability of durum wheat to accumulate Fe was associated with a significantly higher PS release at root level, but the same did not hold true for barley and maize (Celletti et al., 2016a). Interestingly, PS release from wheat roots was also associated with increased S accumulation in both shoot and root tissues (Celletti et al., 2016a).

This aspect of S/Fe interplay highlights new solutions to increase Fe levels in cereal grains and might be exploited for biofortification purposes. Unfortunately, increased Fe accumulation in vegetative tissues resulting from super-optimal S supply did not result in increased Fe concentration in grains, suggesting that the mechanisms involved in the allocation of Fe in seeds might be different from those controlling the root uptake and the allocation in the leaves (Astolfi et al., 2018). However, S over-fertilization allowed plants to at least overcome Fe deficiency (Astolfi et al., 2018).

On the other hand, the reduction of shoot Fe concentration induced by limited S supply was also observed in dicots, such as tomato (Zuchi et al., 2009) and oilseed rape (Muneer et al., 2014). In S-deficient tomato plants it has been ascribed to the inhibition of Fe uptake, due to the prevented induction of the Fe^3+^-chelate reductase and the limited activity and expression of the Fe^2+^ transporter (*IRT1*), also associated to a reduced translocation of Fe to the shoot, as shown by abolished expression of the NA synthase (*LeNAS*) gene (Zuchi et al., 2009). These findings suggested that an adequate S availability is needed to sustain the ethylene (ET) and NA biosynthetic pathways (Zuchi et al., 2009). Quite different behavior on exposure to S deficiency was found for oilseed rape, which exhibited an upregulation of *IRT1* and *FRO1* in the earlier phase. However, the expression of both genes and FCR activity decreased in the later phases, in agreement with Zuchi et al. (2009) (Muneer et al., 2014).

Now, it is well established that the abolished expression of *SlNAS* gene following S deficiency condition effectively resulted in reduced NA levels in tomato (Zuchi et al., 2015). In addition, it has been observed that the two components of the Fe deficiency response (reduction and transmembrane transport) are differentially sensitive to or regulated by ET levels, confirming and extending previous findings in tomato (Zuchi et al., 2009). This is strongly evident considering that ET levels seem to stimulate the components of the Fe deficiency response: the expression of *FIT* (*FER*), the ability to acidify the external medium with regulation of plasma membrane H^+^-ATPase (Lucena et al., 2015), and the reduction of ferric Fe (Romera and Alcántara, 2004). However, increased root ET production following imposition of S deficiency only significantly affected the expression of *SlIRT1* (Zuchi et al., 2015). It has been suggested that S deficiency can result in the induction of the ET pathway (Wawrzynska et al., 2015). Ethylene biosynthesis is indeed related to S through the formation of S-containing metabolites in the S assimilatory pathway such as cysteine (Cys), Met, and *S*-adenosylmethionine (SAM) (Khan et al., 2016). Ethylene and S operate interdependently in regulating plant adaptation processes and abiotic stress tolerance in both optimal and stress environmental growth conditions (Iqbal et al., 2013).

Although these findings account for the hampered capability of dicots to cope with the Fe nutritional disorder under the simultaneous imposition of S deficiency, the question remains, however, whether plant S status and/or S external concentration could modify the capability to take up and accumulate Fe. This challenge has been highlighted recently by Coppa et al. (2018) using a split-root approach, showing that both *SlFER* and *SlFRO1* expression, and Fe^3+^-reducing activity, were induced in the portion of the root system subjected to combined S and Fe deficiency compared to the portion of the root subjected only to Fe deficiency. In addition, it was again confirmed that distinct regulatory processes target *SlFRO1* and *SlIRT1*, since the expression of this latter did not change between the two separated portions of the root system (Coppa et al., 2018). In particular, it was suggested that *SlIRT1* might be controlled by regulatory mechanisms more complex with respect to *SlFRO1* (Coppa et al., 2018).

## Iron Deficiency Modulates Sulfur Uptake and Assimilation Rate

Sulfur is present in soils in different oxidation states (from −2 of sulfide S^2–^ to + 6 of sulfate SO_4_^2–^) (Lewandowska and Sirko, 2008). Plants can use sulfur dioxide (SO_2_) through open stomata but sulfate taken up by roots represents the most important source of S for plants (Marschner, 2012). Sulfate transport across the plasma membrane into the root cells is a secondary active transport system with energy consumption (obtained from ATP hydrolysis) and coupled with protons (at least 3H^+^ per SO_4_^2–^) (Clarkson et al., 1993). Both sulfate uptake from soil and its distribution within plant requires specific transporters, which have been isolated, characterized, and divided into five groups (Buchner et al., 2004).

Once inside roots, sulfate is first reduced and then incorporated into organic compounds (Hawkesford, 2000; Leustek et al., 2000). Both reduction and assimilation mainly occur in leaf tissues since the enzymes involved in these processes are localized in chloroplasts (and, to a lesser extent, in root plastids) (Feldman-Salit et al., 2019). The assimilatory pathway of sulfate starts with sulfate activation, catalyzed by the enzyme ATP sulfurylase (ATPS) which produces adenosine 5′-phosphosulfate (APS), which in turn is reduced to sulfite (SO_3_^2–^) by the activity of APS reductase (APR) with glutathione (GSH) as electron donor. Sulfite is then reduced to sulfide (S^2–^) by the enzyme sulfite reductase (SiR). The first stable compound of S assimilation pathway is the amino acid Cys, synthesized by condensation of *O*-acetylserine (OAS) (produced by the activity of serine acetyl-transferase, SERAT) and S^2–^ in the reaction catalyzed by *O*-acetylserine-(thiol)lyase (OAS-TL) (Hatzfeld et al., 2000; Leustek et al., 2000; Kopriva, 2006; Lewandowska and Sirko, 2008; Koprivova and Kopriva, 2014).

The second S-containing amino acid, Met, is synthesized in three steps using as precursor Cys. In the first step, the enzyme cystathionine-γ-synthase (CGL) catalyzes cystathionine production from Cys and *O*-phosphoserine (OPH), which derives from aspartate. An α, β-elimination of cystathionine is determined by the subsequent activity of cystathionine-β-lyase (CBL), which catalyzes the penultimate step in the biosynthesis of Met, in which cystathionine is cleaved to produce homocysteine (homo-Cys), pyruvate, and ammonia (Ravanel et al., 1998; Saito, 2000). In the last step, a methyl group from N^5^-methyl-tetrahydrofolate (5-CH_3_H_4_PteGlu_n_) is transferred to homo-Cys by the cobalamin-independent Met synthase (MS), producing tetrahydrofolate and Met (Ravanel et al., 1998). Methionine is then incorporated into proteins or converted to SAM by SAM synthetase (SAMS) (Hoefgen and Hesse, 2007; Amir, 2010; Jobe et al., 2019).

*S*-adenosylmethionine is the precursor of ET, biotin, polyamines, NA, and many other secondary metabolites, such as PS. Moreover, SAM is the key substrate of different enzymes and the methyl donor in RNA and DNA modification.

Cysteine is also the precursor of the tripeptide glutathione (γ-glutamylcysteinglycin, GSH), which is synthesized in two ATP-dependent steps (Hoefgen and Hesse, 2007; North and Kopriva, 2007; Takahashi et al., 2011; Jobe et al., 2019). Firstly, γ-glutamylcysteine synthetase (ECS) catalyzes the synthesis of γ-glutamylcysteine (γ-EC) from L-glutamate and L-Cys, then GSH synthetase (GSHS) catalyzes the addition of glycine to the C-terminus to form GSH, which is the main form of reduced S in the phloem. Finally, Cys seems to also be the S-donor for Fe/S-cluster synthesis (Balk and Lobréaux, 2005). Other S-compounds are produced through an alternative pattern in which APS can be phosphorylated by the APS kinase (APSK) with the formation of adenosine 3’-phosphate 5’-phosphosulfate (PAPS), which is used as a precursor by the multiprotein family of sulfotransferases (SOTs) for the synthesis of coumarins, glucosinolates, flavonoids, gibberellic acids, hydroxyjasmonates, phenolic acids, steroids, or sulfate esters (Klein and Papenbrock, 2004; Lewandowska and Sirko, 2008).

Sulfate uptake, translocation, and assimilation require tight and coordinated regulation, which is achieved by both induction and repression. For instance, S deficiency in the growth medium commonly causes an increase in uptake rate, by upregulation of transporters, whereas the accumulation of S-containing compounds in plant cells inhibits uptake rate by downregulation of sulfate transporters (Hawkesford, 2003).

In addition to S availability, other factors are able to positively or negatively modulate S metabolism, such as heavy metals (Astolfi et al., 2004b, 2014; Yamaguchi et al., 2016; Ma et al., 2018).

Recently, it has been highlighted that one of the most striking adaptations to Fe shortage in plants relies on the plant ability to modulate sulfate uptake and assimilation rates (Astolfi et al., 2004a, 2006b; Ciaffi et al., 2013; Paolacci et al., 2014). Overall, Fe deficiency induces a significant increase in the demand for S, and thus activates S uptake and assimilation rate similarly to S deficiency condition. In particular, Fe deficiency increased ^35^SO_4_^2–^ uptake rates by maize and barley roots (Astolfi et al., 2004a, 2006b). Furthermore, Fe deficiency affected the partitioning from the shoots to the roots of reduced S pool within the plant: barley plants exhibited an increased root Cys concentration (Astolfi et al., 2006b), whereas tomato plants showed an increased root-to-shoot translocation of thiols (Paolacci et al., 2014).

Iron availability modulates the expression level of genes involved in both uptake and assimilation of sulfate in grasses, such as barley and durum wheat (Astolfi et al., 2012; Ciaffi et al., 2013), as well as in tomato (Paolacci et al., 2014; Zuchi et al., 2015).

In particular, the expression of the high-affinity sulfate transporter *TdSultr1.3* was significantly induced by Fe deficiency both in shoots and roots of durum wheat plants, as well as most of the genes of the S assimilatory pathway (i.e., *TdATPSul1*, *TdAPR*, *TdSir*, *TdSAT1*, and *TdSAT2*) (Ciaffi et al., 2013). On the other hand, the expression of most of the tomato sulfate transporter genes, belonging to Group 1 (*SlST 1.1* and *1.2*), 2 (*SlST 2.1*), and 4 (*SlST 4.1*), was significantly up-regulated in Fe-deficient roots (Paolacci et al., 2014).

Recently, the dose-response effect in the activation of the adaptive mechanisms in durum wheat has been characterized, and the minimum level of Fe availability (i.e., an Fe concentration threshold) triggering the quite expensive response has been identified. In this respect, it has been demonstrated that there is an Fe-availability threshold (25 μM) below which a complex reorganization of S metabolism (and allocation) is required to guarantee an efficient plant response to the nutritional disorder. This hypothesis is supported by the increase of root ATPS activity, followed by the enhancement of both leaf ATPS and root OASTL activities and the rise of shoot thiols concentrations. It is evident that this process is very expensive and, when activated, could have detrimental consequences on biomass accumulation, thus limiting crop yields as well as weakening the grain quality (Celletti et al., 2016b).

## What Are the Reasons for the Crosstalk Between Sulfur and Iron Nutrition? Are There Common Signals Involved in Their Homeostasis?

**FIGURE 1:**
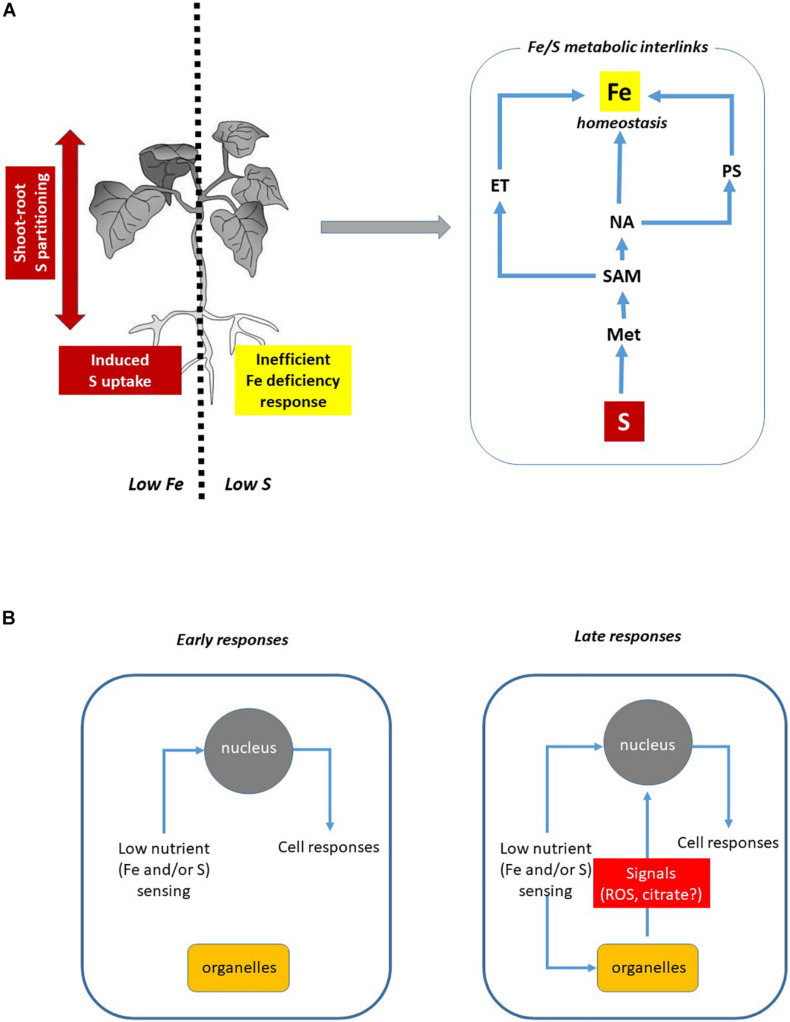
Overview of Fe and S Interaction in plants. **(A)** In plants, Fe and S availability affect S and Fe homeostasis, respectively (left panel, see text for details). The metabolic interlinks between Fe and S seem to be related to the biosynthesis of specific compounds: S is essential for Met synthesis, the pivotal precursor of ET, NA, and PS through the SAM biosynthesis pathway (right panel). **(B)** Hypothetical involvement of organelles (such as mitochondria) in the modulation of Fe and S deficiency-induced responses. The engagement of specific cellular sensing and signaling pathways might depend on the progression/severity degree of nutrient deficiency. Mitochondria impairment might be a source of still unknown signals (likely ROS and/or citric acid) able to modulate cellular responses. ET, ethylene; Met, methionine; NA, nicotianamine; PS, phytosiderophore; SAM, *S*-adenosylmethionine.

The description of both Fe acquisition systems indicates that the amino acid Met could reasonably represent the first connection between Fe and S, since ET, NA, and PS biosynthesis feed on a common precursor, SAM (Hesse and Hoefgen, 2003; Bürstenbinder et al., 2007). The coexistence of traits of both strategies in crossed-form between monocots and dicots (Astolfi et al., 2020) further corroborates this hypothesis (Figure 1A).

Ethylene plays a key role in many processes, such as root hair development, fruit ripening, and seed germination (Wang et al., 2002; Corbineau et al., 2014; Khan et al., 2017). Furthermore, together with jasmonic acid and salicylic acid, ET seems to be involved in abiotic and biotic stress responses (Wang et al., 2002; Yang et al., 2019). More importantly, it has been shown that ET biosynthesis increases under Fe deficiency at root level and is closely correlated with Fe^3+^-reducing capacity (Romera et al., 1999).

Phytosiderophores are secreted by the roots of grasses into the rhizosphere, where PS can form stable complexes with cationic micronutrients, such as Fe^3+^. The most common PS are mugineic acid (MA), deoxymugineic acid (DMA) and epi-hydroxymugineic acid (epi-HMA). Mori and Nishizawa (1987) identified the S-containing amino acid Met as their sole precursor.

The distribution of Fe within the plant is mainly governed by NA (Rudolph et al., 1985; Douchkov et al., 2002; Takahashi et al., 2003).

Based on this evidence, the interplay could be ascribed to a decrease in the production and release of PS induced by S deficiency in grasses, whereas to an impaired ET and NA production in tomato (Figure 1A).

It has been clearly demonstrated that S limited availability hinders plant capability to take up and accumulate Fe by decreasing PS release rate in grasses (Bouranis et al., 2003; Astolfi et al., 2006a; Zuchi et al., 2012; Celletti et al., 2016a) and by inhibiting ET and NA biosynthesis in dicots (Zuchi et al., 2009, 2015). In this respect, and specifically considering grasses, the altered PS release induced by the onset of the xenobiotic detoxification metabolism (Del Buono et al., 2015; Bartucca et al., 2017a, b), which mainly depends on S metabolites, further supports the hypothesis of the relevance of the S pools in plants for an appropriate response to Fe shortage. Furthermore, it has been suggested that limited availability of S could impact the Fe metabolism for its effect on Met synthesis. Under Fe deficiency, Strategy I plants increase ET and NA synthesis (Li and Li, 2004; Molassiotis et al., 2005; Zuchi et al., 2009) with relevant consumption of Met, which represents the precursor of both compounds (Hesse and Hoefgen, 2003), and the same could occur in Strategy II plants, in which Met is the precursor of PS (Guerinot and Yi, 1994). Consequently, the observed regulation of S uptake by roots and root-to-shoot translocation rate might be explained by the need to meet the increased demand for Met and its derivatives in response to Fe starvation (Zuchi et al., 2009, 2015; Paolacci et al., 2014).

However, higher S needs to sustain the activation of Strategy I and II mechanisms cannot fully account for the responses to combined S/Fe deficiency observed in plants.

For example, Fe limitation in wheat plants under adequate S nutrition produced a S deficiency-like response at the molecular level, resulting in higher expression of genes encoding high-affinity sulfate transporters. However, under S deficiency sulfate uptake, capacity of the roots was increased by the upregulation of *TdSultr1.1*, whereas under Fe deficiency it occurred through the upregulation of *TdSultr1.3*, highlighting that the mechanism at the base of the sulfate uptake modulation by Fe or S deficiency might be different (Ciaffi et al., 2013). On the other hand, in tomato plants the transcriptional level of *SlIRT1*, encoding the Fe transporter involved in Fe^2+^ uptake from the soil, in roots exposed to both Fe and S deficiency was approximately seven times higher than in control plants, which corresponded to the sum of the transcript increases observed in the -Fe (five times higher than in control plants) and -S (about two-fold higher than in control plants) plants. This finding suggests that *IRT1* gene expression could be regulated by complex mechanisms that might differ from Fe supply. Several factors regulating the *IRT1* expression at a post-translational level have been identified and characterized in recent years (Shin et al., 2013; Ivanov et al., 2014). In *Arabidopsis thaliana*, *IRT1* acts as metal transceptor critical for optimal Fe and non-Fe metal homeostasis (Dubeaux et al., 2018). In particular, these authors demonstrated that *IRT1* transporters are able to perceive metal excess (for instance Mn and Zn) and to address *IRT1* to protein degradation.

Recently, Robe et al. (2020) showed that, in *Arabidopsis thaliana*, the S deficiency-mediated negative regulation of the Fe uptake machinery might be induced to limit the unspecific transport into the root of potentially toxic divalent cations such as Mn and Zn by *IRT1* (and other transporters like *NRAMP1*). Such findings highlight the importance to investigate the regulation of Fe/S interplay by considering the interaction of both Fe and S also with other essential nutrients.

Thus, it is reasonable to suggest a direct interference of Fe with the signal transduction pathway involved in S metabolism (and *vice versa*), and with the activation of different acquisition strategies.

On the other hand, Fe-S clusters’ assembly has emerged as a further important link between S and Fe (Balk and Pilon, 2011; Mapolelo et al., 2013; Balk and Schaedler, 2014), even if there is no information about the coordination between the assembly of Fe-S clusters and the assimilation pathways of S and Fe. These clusters are the most ubiquitous and versatile prosthetic groups (Pala et al., 2018) and their biogenesis involves three different assembly machineries: SUF machinery located in chloroplast, the Fe-S cluster (ISC) assembly machinery in mitochondria, and the cytosolic Fe-S protein assembly (CIA) machinery located in cytosol (Balk and Schaedler, 2014). In particular, mitochondrion and the ISC has a central role, since this machinery matures all organellar Fe-S protein and provides an unknown S-containing compound, translocated by an ABC transporter into the cytosol, that is necessary for the extramitochondrial Fe-S protein maturation (Stehling and Lill, 2013). However, the evidence that both nutrients are needed for Fe-S cluster biosynthesis, even explaining reduced Fe uptake under S deficiency, does not explain increased S uptake and assimilation under Fe deficiency, suggesting the existence of unknown regulation/signaling mechanisms involved in their close interplay.

All these pieces of evidence seem to support a model in which Fe and S metabolism need to be coordinated, at least up to a certain level, so as to guarantee an adequate formation of the Fe-S clusters. On the other hand, having S metabolism additionally involved in multiple steps of primary and secondary metabolism, an independent form of modulation between the metabolism of the two nutrients, cannot be completely excluded.

At least in part, this hypothesis is mirrored by the intensive overlapping concurrently to a series of distinct responses observed by using metabolome (in tomato, Zuchi et al., 2015) and transcriptome-wide (in wheat, Zamboni et al., 2017) approaches to study the S/Fe interplay.

The potential role of citric acid in plant-adaptation to Fe deficiency and combined S and Fe deficiency has been recently suggested (Coppa et al., 2018; Vigani et al., 2018). The rationale for such an explanation has been that citric acid is produced in mitochondria, where the assembly of Fe-S clusters also occurs (Balk and Pilon, 2011). However, the role of both citrate and retrograde signaling in such regulating process still remains to be elucidated, as recently reviewed by Mendoza-Cózatl et al. (2019). To disentangle the involvement of organelles-nucleus communication in the Fe/S interplay, a broader interaction among nutrients should be considered. Indeed, mitochondria as well as chloroplasts are important cellular sites where complex nutrient interactions take place (Vigani and Hanikenne, 2018; Courbet et al., 2019).

## Concluding Remarks

Identifying a suitable approach to unravel the mechanism underling the Fe/S interplay still remains challenging. The complex regulation of both Fe and S homeostasis involves different regulating pathways with likely different signal molecules (Wawrzyñska and Sirko, 2020). Such complexity relies mainly on the following aspects: (i) different regulating mechanisms might act at local (cell and/or tissue) and systemic level (shoot-root communication); and (ii) nutrient deficiency-induced responses of plants are tailored on the harshness degree of stress.

Due to the high Fe and S demand for both mitochondria and chloroplasts, at the cellular level particular interest has been devoted to the role of retrograde signaling pathway in the regulation of Fe- and S-responsive genes. It has been observed that mitochondrial dysfunction displayed alteration of such genes (Vigani and Briat, 2016). Although some indirect evidence suggested that citrate might be involved in the organelles-nucleus communication, signal molecules involved in such pathway under Fe and S deficiency are still not known. As reviewed by Mendoza-Cózatl et al. (2019), the oxidative stress and, in turn, ROS signaling, might drive retrograde pathway during Fe and S deficiency, since the ROS generated in the *Arabidopsis thaliana* mutants with diverse mitochondrial dysfunction phenotypes might be responsible for the transcriptional reprogramming observed across mutants (Schwarzländer et al., 2012). The transcriptomic analysis of such mutants revealed that the expression of several Fe- and S-responsive genes was affected in plants showing an induced mitochondrial dysfunction (Vigani and Briat, 2016), highlighting that a link between mitochondrial impairment and Fe and S homeostasis would be possible. However, if such regulation occurs directly or indirectly from mitochondria, still remains to be elucidated.

The severity degree of nutrient deficiency also requires different regulating mechanisms depending on stress phases perceived by plants (Vigani and Murgia, 2018). The progression of Fe deficiency perception occurs through three different phases: (i) the alarm stress (referring to the homeostatic control of nutrients content), (ii) the resistance stress (when plant growth starts to be impaired), and (iii) the exhaustion phase (when severe and prolonged deficiency determines growth retardation and death). Such different stress phases involve the engagement of different plant responses. Most likely, such stress progression patterns might also occur for other nutrients, such as S. Therefore, in order to discriminate the molecular actors responsible for Fe/S interplay it is important to identify which step of stress the plants are facing. In this contest, organelles such as mitochondria could play a role in modulation of cellular responses showing different patterns in relation to different severity degrees of nutrient deficiency (Figure 1B).

In conclusion, the analysis of the scientific literature concerning plant mineral nutrition also reported in the present review, clearly shows that the comprehension of the adaptive responses of plants/crops to the nutrient fluctuations in soil have had a notable development in recent years. However, despite the undoubted progress in knowledge, these pieces of information seem to be too limited to fully understand and then to contribute to setting up appropriate agronomical practices for more complex edaphic conditions where more than one single nutrient/element of fertility is concurrently the cause of the (nutritional) stress. Moreover, this aspect appears even more important when the need for an adequate reserve of one nutrient for an appropriate response to the shortage of another one is also considered. A clear example in this sense is represented by Fe and S interplay. The increasing frequency of these cases at the field level, in particular in marginal soils, and the need to rely on all arable land to overcome the challenge of food demand and security, urge researchers to proceed with this methodological approach focused on soil-root system in its integrity and complexity, including the reciprocal interactions among different nutrients. It is definitely complicated but the non-additivity of the adaptive crop responses to each nutrient seems to highlight its strategic nature. Moreover, the transition toward sustainability that characterizes the entire agricultural production system of our time cannot ignore a better and more defined understanding of the phenomena underlying the soil-plant interactions at the rhizosphere that are crucial for the best use of the endogenous nutrient soil resources while preserving its fertility in the long period.

## Author Contributions

SA was responsible for drafting of the manuscript and edited the manuscript. SiC, GV, TM, and StC took part in draft preparation. All authors have read and agreed to the published version of the manuscript.

## Conflict of Interest

The authors declare that the research was conducted in the absence of any commercial or financial relationships that could be construed as a potential conflict of interest.
